# Serotonin and schizophrenia: what influences what?

**DOI:** 10.3389/fpsyt.2024.1451248

**Published:** 2024-10-17

**Authors:** Francisco Jiménez-Trejo, Katia Lorena Jiménez-García, Gustavo Canul-Medina

**Affiliations:** ^1^ Cellular and Tissue Morphology Laboratory, National Institute of Pediatrics, Mexico City, Mexico; ^2^ Faculty of Medicine, Universidad Nacional Autonoma de Mexico, Mexico City, Mexico; ^3^ School of Medicine, Educational Center Rodriguez Tamayo, Merida, Mexico

**Keywords:** serotonin, schizophrenia, neurotransmitters, illness, serotoninomics

## Introduction

Schizophrenia is a chronic and complicated psychiatric and psychological illness. It is characterized by a set of symptoms that include hallucinations, delusions, speech or behavioral disorders, emotional blunting, anhedonia, alogia, apathy, and other defective cognitive abilities. Schizophrenia is a devastating developmental disorder that arises from the interaction between genetic and environmental factors (1). Several lines of evidence suggest that the serotonergic system plays a critical role in the pathogenesis of schizophrenia in at least a subpopulation of patients. According to the neuropathological changes, this heterogeneous condition has been divided into three main domains: positive symptoms, negative symptoms, and cognitive dysfunction. Additionally, some researchers have conceptualized schizophrenia as a disease of functional “dysconnectivity” (2–4) or “synapse disorder” (5, 6), affecting the serotonin quantum release machinery within the synapse during its synthesis, its interaction with its receptors, or its correlation with other neurotransmitters (i.e. dopamine, glutamate, GABA) (7, 8). Acute relapses of this disease are characterised by the exacerbation of positive psychotic symptoms, while chronic disabilities occur due to cognitive causes and negative symptoms (apathy and social withdrawal) (9). The disease profoundly affects not only the individuals who suffer from it but also their families and caregivers, typically manifesting during late adolescence or early adulthood (1, 9).

Because the condition is so heterogeneous, the presence of symptoms from these domains in the disease makes its diagnosis and treatment difficult. The development of some mental diseases, including schizophrenia, are marked by subtle molecular, heritable, and epigenetic changes, which initiate a cascade of events within individual cells before overt signs and symptoms manifest themselves. These events include effective quantum release; appropriate signaling by receptors and activation by second messengers; the functionality of transporters; the ability to take up, store, and release serotonin; the presence of isoforms; the activity of tryptophan hydroxylase-2 (TPH2; lack of activity); the availability of the amino acid L-tryptophan (L-Trp); deviation towards other pathways of its metabolism how to as the kynurenine-like pathway; mutations; and other dysfunctions. Therefore, individuals with schizophrenic parents or siblings will have a higher risk of developing the disease due to this hereditary component (between 8% and 12% (10). The most common theory regarding the neurobiology of schizophrenia is a chemical imbalance in the brain, marked by deregulation of the mesolimbic pathway as a result of dopaminergic hyperactivity and, in some cases, alterations in the serotonergic system in specific nuclei of the brain (prefrontal cortex, temporal cortex, thalamus, hypothalamus, and basal ganglia) or in the interaction between dopamine and serotonin (9).

Serotonin is a molecule with numerous functions within the central nervous system (CNS), which is why it plays an essential role in the delicate balance of neuronal function. Alterations in serotonin concentration can contribute to the onset of schizophrenia, and conversely, schizophrenia can gradually affect the serotonergic systems of the brain (11). As the initial molecular and genetic changes progress, often silently and insidiously, these molecular perturbations extend to higher organizational levels: tissues, organs, and systems. In this way, all of these changes will culminate in dysregulations that will contribute to the characteristic symptoms of schizophrenia (i.e. altered perception) (12). A key limitation in the study of schizophrenia is that it is difficult to conclusively determine causation, given the interactive influences exerted by the different molecular and physiological mechanisms involved (9, 11, 12). Therefore, the understanding of these early molecular events is essential for unravelling schizophrenia’s etiology, including neurotransmitter dysregulation, neuroinflammation, the hypothalamic–pituitary–adrenal (HPA) axis, the gut–brain axis (GBA), oxidative stress, and mitochondrial dysfunction and thus for developing interventions that could slow down or modulate these processes before symptomatic expression. In recent years, the gut microbiota (GM) has emerged as another important factor in the development of neurological–psychiatric disorders (including schizophrenia), with changes in the composition and quality of individual microbial communities potentially having serious health consequences. This cerebral–gut axis is a two–way communication pathway, in which brain–gut communication occurs through the autonomic nervous system (ANS), mainly the vagus nerve. Research has revealed that approximately 90% of the impulses within the cerebral–intestinal axis are transmitted centripetally —from the intestines to the brain—and only 10% centrally. In this respect, certain intestinal bacteria, such as Escherichia spp., Candida spp., and Enterococcus spp., produce various substances, including serotonin, involved not only in communication within the intestinal microflora but also in systemic and peripheral effects that influence brain function (13).

The reality is that there is a close mutual relationship between serotonin and schizophrenia, and this bidirectionally influences the way in which serotonin manifests itself in this mental illness. But which one comes first: changes in the serotonergic system or schizophrenia? This is an important question to which we seek to provide an answer in this occasion.

## Hierarchy of action versus manifested symptoms

Since its discovery as a molecule by Page et al. in 1948 (14), serotonin (5–hydroxytryptamine, 5–HT, C_10_H_12_N_2_O) has led to great expectations and interest due to the implications it may have for various pathologies, including those related to mental health such as schizophrenia. This disease affects millions of people around the world, making it a main challenge for the community of doctors and scientists who seek to reduce its effects and improve the quality of life of patients. Generally, the disease begins without presenting symptoms in an absolute and pathognomonic manner, unless the disease has a genetic, immune–related, or acutely toxic origin. The body can warn of the disease before the patient perceives any specific signs and symptoms, or it can be recognized through the information provided by the patient’s family, so that a diagnosis can be established and specific treatments can be initiated (5, 6).

Like most psychotic diseases, schizophrenia is considered to be caused by an imbalance in neurotransmitters (e.g. dopamine [DA], glutamate, GABA, and serotonin). The simple model of abnormally high DA transmission could not explain the pathophysiology of schizophrenia or the therapeutic actions of antipsychotic medications. Nonetheless, this model has persistently served as the primary explanation for schizophrenia (1). Due to the complexity of its neuropathology, schizophrenia is a complicated disorder The lack of understanding of its pathophysiology and its different forms and modes of presentation in patients often leads to it being unmanageable as a disease within clinical settings (15).

The global prevalence of schizophrenia is around 1%–1.5%. Between 1990 and 2019, the number of people with schizophrenia increased from 14.2 to 23.6 million (16, 17). Its etiological origin is heterogeneous, and the interaction between genetic (heritability), social (isolation, discrimination), psychological (school bullying, aggressive abuse), and environmental (exposure to metal toxicity) factors complicates etiological and diagnostic counts. However, when any of these interactions occur, it is reflected in many patients, even generating associations with other psychiatric conditions such as depression. Therefore, this comorbidity suggests a potential common pathophysiology and/or common genetic predictors of these mental disorders. Mesolimbic dopamine release in patients experiencing hallucinations or psychotic symptoms is known to cause increased activation of the thalamus, hippocampus, and striatum in the CNS. The hyperactivity of the hippocampus appears to be driven by a loss of parvalbumin–containing gamma–aminobutyric acidergic (GABAergic) interneurons, a phenomenon observed in postmortem analyses of schizophrenia brains.

The molecular mechanisms linking schizophrenia, depression, and other psychiatric diseases are polygenic. Currently, genes candidates under study are *GRIN1*, *GPM6A*, *SEPTIN4*, *TPH1*, *TPH2*, *CACNA1C*, *CACNB2*, *BCL9*, and *C4A* (18). For this last gene, Sekar et al. (2016) postulated that excessive activity of *C4A* during the development of schizophrenia might explain the reduction in the number of synapses in the brains of affected individuals. This gene is associated with the major histocompatibility complex (MHC) locus, which represents the strongest genetic association of schizophrenia at the population level (19). Other genes are probably involved in schizophrenia, and the complexity of responses to 5–HT may involve other factors, so further studies should be performed for a genetic diagnosis.

More recently, Kossatz et al. (2024) contributed to unravelling the role of 5–HT_2A_–mediated pathways in schizophrenia–like behavioral responses by applying a multidisciplinary approach, integrating computational modelling, *in vitro* and *in vivo* experiments, and postmortem human brain studies. Since this receptor is a prominent target for the treatment of schizophrenia, they demonstrated that memory deficits are regulated via Gαq protein activation, whereas psychosis–related behavior is modulated through Gαi1 stimulation (20).

Additionally, recent advances in animal models seek more successful therapeutic approaches within clinical settings related to pathophysiology, such as dopamine, glutamate, GABA, and serotonin. These animal models are based on the release of neurotransmitters, pharmacological interactions, processes during neurodevelopment, and genetics. In schizophrenia, the presence of gene polymorphism has also been reported (e.g., dysbindin, *DICS1*, and *NRG1*). Several hypotheses have been based on dopamine, glutamate, and serotonin, which have served as successful models within schizophrenia studies. Pharmacological therapies have even been designed based on these models, and many of them are still used today (21).

New targets, such as the orexin system, the muscarinic and nicotinic receptors, and the cannabinoid receptors, have been addressed with the aim of alleviating negative and cognitive symptoms. Additionally, non–pharmacological models have been developed, such as the post–weaning social isolation model (maternal deprivation) and the isolation parenting model, with the aims of imitating the symptoms of schizophrenia and creating and testing new approaches to drug therapy, constituting a great advance in treating psychiatric disorders. Different behavioral tests have been evaluated in these specific models. However, while animal models are inherently limited and can only approximate the pathological state observed in humans, if they are based on clinical observations, they are essential for providing insights into the mechanisms of action of novel drug candidates (22). Thus, better translational models and behavioral tests for psychological disorders, such as schizophrenia and related psychiatric conditions, should be more abundant and available (15).

Furthermore, it is important to promote the practical concept of serotoninomics to contribute to the search for precise answers in basic, clinical, and translational research, as well as in clinical pharmacology, particularly concerning mental illnesses such as schizophrenia and/or depression. Our group proposed this concept seeking to encompass all studies focused specifically under the topic of serotonin and its associated system. This includes experimental techniques and laboratory tools that have been used since its discovery until today (23, 24). Adopting this approach can help us in elucidating causal links within a health state, such as alterations of the serotonergic system itself, and the alterations that it produces within the organs or tissues in which it participates, as well as the wide range of biological systems in which it is present.

Therefore, assessing the scope and limits of our opinion means analyzing, for example, whether the psychotic alterations of schizophrenia in some types of patients, given their characteristics shown, are triggered by alterations in the serotonin system and constitute an ultimate explanation. Thus, secondary consequences will almost always follow psychiatric manifestations, which in this case correspond to patients who attend mental health clinical consultations.

On the other hand, confinement, social distancing, and isolation due to the SARS–CoV–2 (COVID–19) pandemic increased psychological stress and affected people’s mental health (25, 26). Realizing that SARS–CoV–2 or other opportunistic microorganisms (e.g. bacteria, viruses, prions, or fungi) can enhance mental health problems in humans is important for combating their effects, which increase as mental conditions are altered (26, 27).

## Conclusion

As proposed by Shnayder et al. (18), the planning and performance of associative genetic studies in psychiatric diseases, such as schizophrenia, and the determination of the complete genome of comorbid diseases will be important for the development of gene–based therapies, as well as for clinical predictors based on therapeutic strategies within personalized medicine. These steps must be carried out despite the current problems of predictive and personalized psychiatry in the diagnosis and treatment of comorbid diseases, which are still far from being resolved more than 76 years after the discovery of serotonin. Diagnoses and clinical approaches must be urgently improved to define what type of psychiatric condition the patient presents in order to quickly determine the best pharmacological therapy to reduce their symptoms and abnormal behaviors. Understanding these early molecular events is essential for unravelling the etiology of schizophrenia and for developing interventions that can slow down or modulate these processes before symptomatic expression. Furthermore, systematic reviews (qualitative or quantitative reviews and meta–analysis) and/or bibliometrics may help guide the pharmacological approach, therapeutic decisions, and the continuity of treatment, including the facilitating role of artificial intelligence in human medicine.
